# Influence of the Number of Sets at a Strength Training in the Flexibility Gains

**DOI:** 10.2478/v10078-011-0058-1

**Published:** 2011-10-04

**Authors:** Roberto S. Júnior, Thalita Leite, Victor M. Reis

**Affiliations:** 1 Research Centre for Sport Sciences, Health and Human Development (CIDESD), Vila Real, Portugal; 2 Rio de Janeiro Federal University, Physical Education Post-Graduation Program, Rio de Janeiro, Brazil; 3 Department of Sport Sciences, Exercise and Health, University of Trás-os-Montes and Alto Douro (UTAD), Vila Real, Portugal

**Keywords:** resistance training, training volume, stretching

## Abstract

The aim of this study was to investigate the effects of 10 weeks of strength training with different number of sets and their influence on flexibility of young men. Sixty men were divided into three groups as follows: group that trained 1 set per exercise (G1S), group that trained 3 sets per exercise (G3S) and control group (CG). The training lasted 10 weeks, totaling 30 training sessions. The training groups performed 8 to 12 repetitions per set for each exercise. The flexibility at Sit and Reach Test was evaluated pre and post-training. Both trained groups showed significant increase in flexibility when compared to pre-training and the G3S showed significant difference when compared to CG post-training. According to this study, the strength training carried out without flexibility training promotes flexibility gains regardless the number of sets.

## Introduction

According to the American College of Sports Medicine (ACSM, 2000) the physical fitness is related to health through five basic components: body composition, aerobic capacity, strength, endurance and flexibility. Among these components, strength and flexibility are important variables of physical fitness, and their appropriate levels are required not only for the health promotion and maintenance, and functional autonomy but also for safe and effective participation in sports (ACSM, 1998).

Regular practice of strength training can be aimed to increase flexibility. Several studies found that isolated strength training promotes improvement in flexibility (Barbosa et al., 2002; Fatouros et al., 2002; Fatouros et al., 2005; Fatouros et al., 2006; Monteiro et al., 2008; Santos et al., 2010; Simão et al., 2011). Fatouros et al. (2002) investigated the influence of aerobic training, strength training and the combination of both in range of motion of elderly sedentary men, 65–78 years. In the group that only trained strength, significant differences were found for all the joints that were assessed. Barbosa et al. (2002) investigated the effects of 10 weeks of strength training on the flexibility response of elderly sedentary women, 62–73 years. Flexibility was assessed using the Sit and Reach Test, before and after strength training. They found that the training caused a significant increase in flexibility, while no difference was found in the control group. Nóbrega et al. (2005) investigated the interaction between strength training and flexibility in sedentary young adults. After 12 weeks, the authors found that isolated strength training was not able to increase the flexibility significantly. Monteiro et al. (2008) verified the effect of strength training on flexibility in sedentary women, and the strength training program was implemented through circuit training. The results showed different behaviors for different joints and movements before and after the training period. In conclusion, the results suggested that strength training increased flexibility in sedentary adult women. Recently, Simão et al. (2011) verified the flexibility gains in 80 sedentary women divided into four groups. In the group that only performed strength training (n=20) for 8 exercises for the upper and lower body, they performed 3 sets of each exercise, in a periodized form, was verified the flexibility improvement in the Sit and Reach Test.

In sum, the literature reports the positive effects of strength training on flexibility and the available studies used different types of training methods and different combinations of exercises (Barbosa et al., 2002; Fatouros et al., 2002; Fatouros et al., 2005; Fatouros et al., 2006; Monteiro et al., 2008; Santos et al., 2010; Simão et al., 2011). However, to our knowledge, no study has investigated whether the number of sets could affect the changes in flexibility. Therefore, the purpose of this study was to investigate the effects of 10 weeks of strength training with different number of sets, and its influence on flexibility.

## Material and Methods

### Subjects

Sixty men, intentionally chosen, were divided into three groups: 1 set per exercise (G1S) (n=20), 3 sets per exercise (G3S) (n=20) and control group (CG) (n=20). To be included in the study, all participants must have the following characteristics: a) be recreationally trained in strength training and flexibility at least one year before the study began; b) not perform any type of regular physical activity other then the prescribed; c) not present any functional limitations to strength training or for the performance of the tests involved in the study; d) not present any medical limitations that could influence the training program, and; e) not use any nutritional supplementation (the military diet was the same for all participants). All participants signed an informed consent form that explains the testing and training procedures conducted during the study. The study protocol was approved by the “Research Ethics Committee of Rio de Janeiro Federal University (Brazil)”.

Data collection pre and post-training (10 weeks) was performed in four days. On the first visit, between 7:00 and 8:00 am, anthropometric and flexibility measures were made. On the second day (24 hours after), all the tests were repeated to determine the test retest reliability. On the third day (24 hours after), the 5 repetitions maximum test (5RM) was applied. On the fourth day, 48 hours later, the 5RM test was repeated.

### 5 Repetitions Maximum Test (5RM)

The subjects were evaluated in two non-consecutive days in both pre and post training. The 5RM test was conducted for the exercises: bench press (BP) and leg press (LP). All exercises were performed on machines Rotech Fitness® (Goiânia - Brazil). To minimize the error during the 5RM tests, the following strategies were adopted (Simão et al., 2005): (a) standardized instructions concerning the testing procedures were given to participants before the test; (b) participants received standardized instructions on exercise technique; (c) the exercise technique of subjects was monitored and corrected as needed during testing, because variations in the positioning of the joints involved in the movement could activate other muscles, leading to misinterpretation of scores, and; (d) standard verbal encouragement was provided during the test procedure. During the 5RM test, each subject had a maximum of three attempts at each exercise with a rest interval of 5 minutes between them and 10 minutes before the start of the test of the next exercise. The standard exercise technique was conducted for each exercise. No pause was allowed between the eccentric and concentric phases of a repetition nor between the repetitions. The range of motion determined should be achieved to define completion of a successful repetition. The heaviest load achieved on either of the test days was considered the 5RM.

### Flexibility Measurement (Sit and Reach Test)

Flexibility was measured before and after 10 weeks using a Sit and Reach Test (ACSM, 2000). The subject sat with their feet firmly against the testing box, keeping their knees extended and hands placed one over the other; reached forward, sliding the hands along the measuring ruler. The considered score was the greatest distance recorded in the three attempts with a 10-second interval between them (ACSM, 2000). The same procedure was performed after training. All flexibility tests were conducted in the same period of the day. Data collected during the first evaluation were not available to the examiner to prevent information bias during the measurements taken after training.

Before the flexibility test, a warm-up of 4 stretching exercises was performed for the muscle groups involved in the evaluation. Two sets of static stretching were used for the warm-up protocol, holding the position for 10 seconds in each set, until a point of slight discomfort was reached. A 10-second interval was provided between the warm-up stretching sets. The warm-up exercises followed the protocol of the Sit and Reach Test of American College of Sports Medicine (2000).

### Training Protocol

The exercise order for all groups (G1S and G3S) was: BP, LP, lat pull-down, leg extension, shoulder press, seated leg curl, biceps curl, abdominal crunch and triceps extension. The CG not participated in the strength training program. All subjects performed the sets with moderate intensity (8 to 12 repetitions) in each exercise. During the sessions, the subjects were verbally encouraged to perform all sets to concentric failure and the same definitions of a complete range of motion were used to define completion of a successful repetition. There was no attempt to control the velocity of the repetitions performed. The training load was increased when the individual could perform more than the prescribed number of repetitions (12 repetitions). Frequency of the training program was 3 sessions per week with at least 48 to 96 hours between the sessions. A total of 30 sessions was performed during the training period. Prior to each training session, the subjects performed a specific warm-up, consisting of 10 repetitions with approximately 50% of the load used in the first and second exercises of the training session. Adherence to the strength training was 100% for all participants. All training sessions were monitored by an experient physical education professional and the subjects were not allowed to perform aerobic or flexibility exercises during the training period.

### Statistical Analyses

All data were expressed as mean ± standard deviation. Statistical analysis was initially performed by Kolmogorov-Smirnov normality test and for the homocedasticity test (Bartlett criterion). All variables showed normal distribution and homocedasticity. An ANOVA one-way was used and, when the differences were significant, the Tukey post hoc test was applied for comparisons. An alpha level of p<0.05 was considered statistically significant for all comparisons. Statistica® 6.1 statistical software was used for all statistical analysis.

## Results

**Figure 1:**
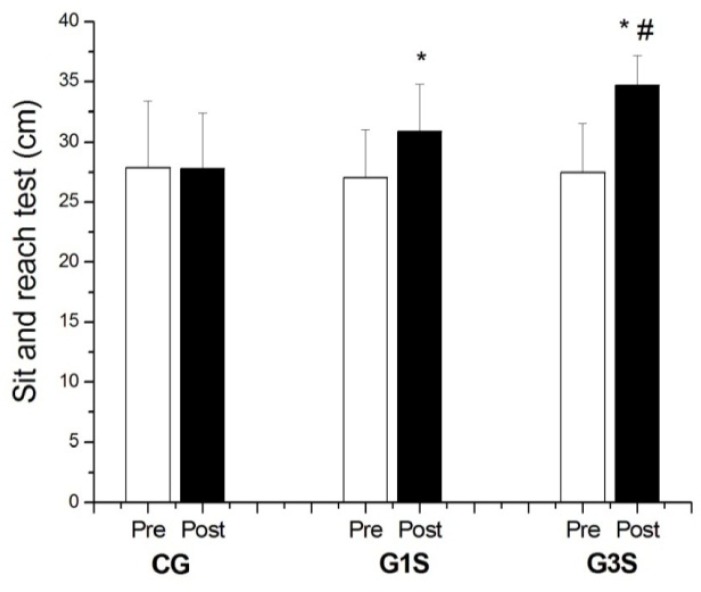
Sit and Reach Test result. Values expresses as mean ± standard deviation. * Statistically significant difference compared to the pre-training. # Statistically significant difference compared to CG post-training (p<0.05)

The trained groups showed a significant increase in flexibility in relation to pre-training and the G3S showed significant difference when compared to CG in the post-training (Figure 1).

Table 1 shows that groups were not statistical different when compared between them, both in the pre- and post-training in anthropometric measures. Table 2 shows the strength gains in 5RM.

## Discussion

The purpose of this study was to examine, in young recreationally trained men, if the isolated strength training in different training volumes (1 or 3 sets per exercise) could influence in flexibility gains. The main finding of this research was that the strength training performed without the flexibility training promotes flexibility gains regardless of the number of sets. In fact, both trained groups (G1S and G3S), promoted significant gains post-training when compared to pre-training, but only G3S showed significant gains when compared to CG post-training. Regard to strength gains in 5RM, it was observed that G3S showed greater gains in both the analyzed exercises.

Actually, only two studies (Fatouros et al., 2006; Santos et al., 2010) investigated the influence of methodological variables of strength training on flexibility gains. Santos et al. (2010) showed that different training methods, alternated strength training and agonist/antagonist, were able to significantly increase the flexibility levels after 8 weeks of training. The study was conducted in sedentary young women (24 to 28 years) and lasted for 8 weeks. Eight exercises were conducted for entire body, in three weekly sessions. The flexibility measure was conducted through goniometry and flexibility gains were showed regardless of the training method used. The authors concluded that strength training can increase the flexibility levels of an individual. Fatouros et al. (2006) showed that regardless of intensity of strength training, 40, 60 or 80% of 1RM, the flexibility increased after 6 months of training. The study was conducted in elderly untrained (65 to 78 years) and lasted six months. The subjects performed 10 exercises for the entire body, three times weekly. The trunk flexibility measure was performed through the Sit and Reach Test and was showed that flexibility gains were dependent of training intensity. In the groups that trained at 40, 60 or 80% of 1RM, flexibility gains in Sit and Reach Test were 13, 22 and 26%, respectively. In addition to the Sit and Reach Test for the trunk measure, the goniometry was used for measures in other joints, but the results were similar to that obtained in the Sit and Reach Test, that is, flexibility gains were greater when groups trained at moderate intensity (60% of 1RM) or at high intensity (80% of 1RM) when compared to the group that trained at lower intensity (40% of 1RM). Authors concluded that flexibility gains are dependents of strength training intensity.

Despite of methodological differences between our study and those conducted by Santos et al. (2010) and Fatouros et al. (2006), our results allow us to infer that volume of strength training also appears to influence in flexibility gains. We used nine strength exercises, but alternating upper and lower body for 10 weeks, three times a week. Perhaps, the greatest difficult in finding significant differences between groups that trained 1 or 3 sets was the fact that both were recreationally trained in strength training and flexibility. Maybe if the training duration was longer, we would find any difference in the Sit and Reach Test. This observation is due to the fact that the group which performed 3 sets had a significant flexibility gain in relation to CG post-training. The same not happen with the 1 set group in relation to CG post-training. Thus, we believe that the training volume of strength training in trained people influences the flexibility gains, but it is necessary to conduct a study with longer duration.

Concerning the flexibility gains through the isolated strength training, our results corroborate with previously published studies in this area (Barbosa et al., 2002; Fatouros et al., 2002; Fatouros et al., 2005; Fatouros et al., 2006; Monteiro et al., 2008; Santos et al., 2010; Simão et al., 2011). However, only one study (Simão et al., 2011) verified the isolated strength training compared to isolated flexibility training and the combination of both. Eighty young women were divided into four groups: strength (n=20), flexibility (n=20), strength and flexibility (n=20) and control group (n=20). The flexibility measure was done through the Sit and Reach Test and, they found that the group that trained strength and flexibility in the same training session had greater flexibility gains than the group that trained only strength or only flexibility. Interestingly, the group that trained strength and flexibility in the same session had a longer training compared to the groups that only trained strength or flexibility. This leads us to believe that the total training volume seems to affect the flexibility gains, but it is needed future studies with this purpose in special for really infer this conclusion.

In relation to strength gains in 5RM for bench press and leg press, it was evident that multiple sets promoted greater strength gains. Although it was not the main focus of our experiment, we found that strength gains are dependent of training volume and it is important to observe that both, the flexibility and the strength gains seem to be dependent on the training volume. Our findings regarding strength gains corroborate with the previously showed in the literature, that is, there is a dose dependency relationship in strength gain related to the number of sets (Rhea et al., 2002; Rhea et al., 2003; Bottaro et al., 2009).

When think in health gains or physical performance, it seems to perform isolated strength training, with a greater volume of training, is sufficient to promote flexibility gains. Health professionals can use this training strategy to increase flexibility when the session time is reduced. However, it is important to emphasize that only the isolated strength training might not be enough to increase flexibility in a specific way.

## Figures and Tables

**Table 1:** Anthropometric measures pre and post-training in the two groups of subjects

	Height (cm)	Body Mass (kg)	

		Pre	Post
CG	175.68 ± 4.39	78.39 ± 5.68	78.56 ± 4.39
G1S	177.64 ± 2.57	78.19 ± 3.52	80.16 ± 3.00
G3S	178.22 ± 2.71	79.02 ± 4.85	82.31 ± 4.36

**Table 2:** 5-RM values pre and post-training in the two groups of subjects

BENCH PRESS		

	Pre	Post
CG	72.00 ± 6.81	72.28 ± 6.35
G1S	71.11 ± 8.20	76.56 ± 8.90
G3S	71.67 ± 8.31	82.22 ± 5.57*

LEG PRESS		

	Pre	Post
CG	166.40 ± 14.20	168.4 ± 16.04
G1S	166.60 ± 15.74	184 ± 12.12*#
G3S	168.82 ± 14.19	204.6 ± 13.53*#@

* Difference compared to pre-training.

# *Difference compared to CG post-training (p<0.05)*.

@ Difference compared to G1S post-training (p<0.05).
